# Temporal autocorrelation in host density increases establishment success of parasitoids in an experimental system

**DOI:** 10.1002/ece3.1505

**Published:** 2015-06-18

**Authors:** Elodie Vercken, Xavier Fauvergue, Nicolas Ris, Didier Crochard, Ludovic Mailleret

**Affiliations:** 1 INRA, UMR 1355 Institut Sophia Agrobiotech06900, Sophia Antipolis, France; 2 Université Nice Sophia Antipolis, UMR 7254 Institut Sophia Agrobiotech06900, Sophia Antipolis, France; 3 CNRS, UMR 7254 Institut Sophia Agrobiotech06900, Sophia Antipolis, France; 4 INRIA, Biocore06902, Sophia Antipolis, France

**Keywords:** Environmental noise, introduced populations, propagule pressure, red noise, transitory dynamics, *Trichogramma*, white noise

## Abstract

Environmental variation is classically expected to affect negatively population growth and to increase extinction risk, and it has been identified as a major determinant of establishment failures in the field. Yet, recent theoretical investigations have shown that the structure of environmental variation and more precisely the presence of positive temporal autocorrelation might alter this prediction. This is particularly likely to affect the establishment dynamics of biological control agents in the field, as host–parasitoid interactions are expected to induce temporal autocorrelation in host abundance. In the case where parasitoid populations display overcompensatory dynamics, the presence of such positive temporal autocorrelation should increase their establishment success in a variable environment. We tested this prediction in laboratory microcosms by introducing parasitoids to hosts whose abundances were manipulated to simulate uncorrelated or positively autocorrelated variations in carrying capacity. We found that environmental variability decreased population size and increased parasitoid population variance, which is classically expected to extinction risk. However, although exposed to significant environmental variation, we found that parasitoid populations experiencing positive temporal autocorrelation in host abundance were more likely to persist than populations exposed to uncorrelated variation. These results confirm that environmental variation is a key determinant of extinction dynamics that can have counterintuitive effects depending on its autocorrelation structure.

## Introduction

Understanding the factors that explain the success or failure of introductions has been a major challenge for ecologists over the last decade, with potential applications in the fields of invasion biology, conservation biology, and biological control. A striking feature of the introduction process is that the majority of populations introduced into a novel environment go extinct within a few generations and never establish (Williamson 1996; Seddon et al. 2007; Simberloff 2009). This phenomenon was observed not only in the case of accidental introductions (Williamson 1996; Booth et al. 2003) but also for planned introductions (Freckleton 2000; Noël et al. 2011) where establishment may fail despite intense efforts to maximize establishment success. Among the potential factors underpinning establishment failures, environmental variability is expected to be both ubiquitous and uncontrollable and, as such, is a likely candidate to explain a significant part of early extinction events (Fauvergue et al. 2012).

Environmental variation is expected to affect extinction probability in two ways. First, in the case of catastrophic events such as storms, droughts, or fires, an entire population can be wiped out almost instantly (Shaffer 1987; Lande 1988). Second, even in less dramatic circumstances, lower magnitude environmental variability can drive population-level fluctuations in vital rates, such as mortality and fecundity (Engen et al. 1998; Lande et al. 2003). This, in turn, can increase population variability and thereby increase the probability that the population will hit low population sizes where they are vulnerable to extinction (Shaffer 1987; Lande 1988; Reed 2005; Fauvergue et al. 2012). In the case of introduced populations, theoretical models and experimental data confirm that establishment probability should decrease in the presence of environmental variation (Grevstad 1999a; Drake and Lodge 2004; Drury et al. 2007).

In these studies, environmental variation is considered purely stochastic, that is, the successive states of the environmental variable considered are independent draws from a random variable. However, most environmental factors in nature display some degree of temporal autocorrelation (Halley 1996; Vasseur and Yodzis 2004), which is expected to affect population growth and persistence quite differently from uncorrelated variation (Johst and Wissel 1997; Morales 1999; Holt et al. 2003; Schwager et al. 2006). In particular, positively autocorrelated environmental variation should increase population persistence in two specific situations. First, in systems exposed to catastrophes, the probability of a rare, extreme event occurring is lower when environmental variation is positively autocorrelated, because successive states of the environment resemble each other closely and environmental variation is globally smoother (Heino et al. 2000; Schwager et al. 2006). A positive effect of positively autocorrelated environmental variation is also expected when population dynamics are overcompensatory (i.e., the costs of competition increase at higher densities, Gilpin and Ayala 1973; Hassell 1975) and the environmental variation affects carrying capacity. In overcompensatory dynamics, populations exceeding carrying capacity suffer from severe population crashes that might result in extinction (Allen et al. 1993; Costantino et al. 1997; Ripa and Lundberg 2000). Yet, when capacity is autocorrelated, the quantity of offspring produced at a given generation will on average be close to the carrying capacity at the next generation, so that the density dependence feedback will tend to remain moderate. In this case, temporal autocorrelation in environmental stochasticity tends to reduce extinction risk (Petchey et al. 1997; Schwager et al. 2006). Therefore, in the case of an introduced population, environmental variation can be expected to affect establishment probability either negatively or positively, depending on the structure of the variation (uncorrelated or autocorrelated) and the characteristics of population dynamics.

Hymenopteran parasitoids are widely used as biological control agents and as such represent a well-known group for the study of introduced populations (Grevstad 1999b; Marsico et al. 2010; Fauvergue et al. 2012). For parasitoids, a major component of the environment is the abundance of their hosts, which is known to respond to a fine-tuned dynamical interaction between both species. From the parasitoid perspective, this interaction is an intrinsic source of environmental variability: In a system that is not at equilibrium, like in the first generations after parasitoid introduction or during sustained oscillatory dynamics, abundances of hosts and parasitoids are expected to fluctuate even if other components of the environment are constant (May et al. 1988; Kidd and Amarasekare 2012). This may result in temporal autocorrelation in host abundance during the parasitoid establishment phase (Sait et al. 2000; Kidd and Amarasekare 2012; Ruokolainen 2013). In contrast, external factors unrelated to host–parasitoid dynamics (e.g., climatic factors, interactions with other species, and habitat disturbance) should rather induce uncorrelated (or more weakly correlated) fluctuations in host abundance.

**Figure 1:**
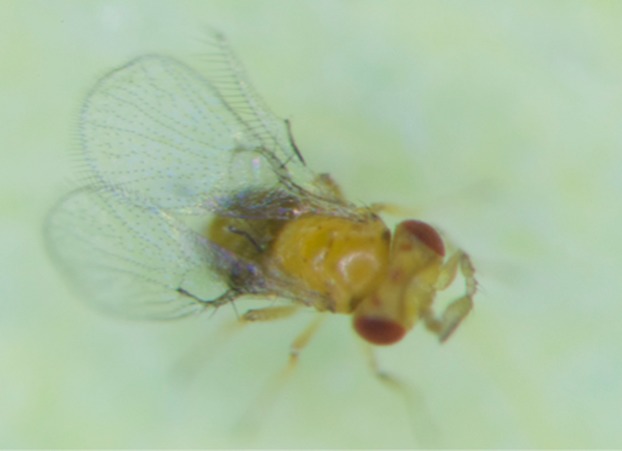
Female *Trichogramma chilonis*. Real size = 1 mm.

To better understand how the structure of environmental variation impacts population establishment, we investigated the respective influence of external, uncorrelated variations in carrying capacity versus variations generated by the host–parasitoid interaction on the dynamics and persistence of introduced parasitoid populations in an experimental system. More specifically, we tested the predictions that uncorrelated environmental variation decreases population size (Benton et al. 2001) and increases population variability and extinction probability (Drake and Lodge 2004). In contrast, as parasitoids are sensitive to intraspecific competition and may display overcompensatory dynamics (Taylor 1988), we hypothesized that autocorrelated variations in host abundance induced by host–parasitoid coupled dynamics would result in higher persistence than uncorrelated, purely random variations. We tested these predictions using laboratory populations of the parasitoid wasp *Trichogramma chilonis* (Fig.1) and its factitious host *Ephestia kuehniella* during the first ten generations after parasitoid introduction. Establishment dynamics were described by parasitoid abundance at each generation, its coefficient of variation along each time series, and the occurrence of parasitoid extinction events. Temporal autocorrelation in host abundance was manipulated either by assigning host abundance randomly from a uniform distribution, or by mimicking the natural host–parasitoid feedback loop, where the high consumption rate of hosts by parasitoids at one generation results in fewer hosts available for parasitism in the next generation. We chose to manipulate host fluctuations indirectly rather than setting different levels of temporal autocorrelation in order to address specifically the impact of fluctuations driven by internal host–parasitoid dynamics in comparison with purely random variations. These experimental treatments on host dynamics were crossed with different initial population sizes for parasitoids. Indeed, the number of introduced individuals is known to influence establishment success in this experimental system (Vercken et al. 2013) and might interact with environmental variation as the deleterious effects of low population size are often most apparent in unfavorable environments (Griffen and Drake 2008; Hufbauer et al. 2013). Finally, the experiment was replicated for two different geographic strains to estimate the influence of genetic background on the relationship between the structure of environmental variation, population size, and establishment dynamics.

## Material and Methods

### Study system

We used the wasp *Trichogramma chilonis* (Hymenoptera: Trichogrammatidae) as a model species (Fig.1). *Trichogramma* are minute solitary parasitoids of Lepidopteran eggs widely used as biological control agent against noxious species (Smith 1996). The widespread species *T. chilonis* is more specifically released against the sugar cane spotted borer *Chilo sacchariphagus* (Tabone et al. 2010). In addition, *Trichogramma* have consistently been used as model species in behavioral ecology (Wajnberg et al. 2000; Boivin et al. 2004; Huigens et al. 2010; Martel et al. 2010; Kruidhof et al. 2012) and population biology (Vercken et al. 2013).

Two bisexual strains of *T. chilonis* were used for this experiment. Sex determinism is explained by arrhenotoky, fertilized and unfertilized eggs, respectively, developing into diploid females and haploid males (Quicke 1997; Heimpel and de Boer 2008). The two strains were founded from individuals caught in the field in Taiwan (in 1987) and Reunion Island (in 1998, see more details in Benvenuto et al. 2012; Vercken et al. 2013). The strains were then maintained in laboratory conditions on the factitious host *Ephestia kuehniella* (flour moth). For this experiment, temperature and light conditions were set on a cycle of 16-h daylight (25°C)/8-h dark (20°C) with constant 70% humidity. Under these conditions, generation time was 9 days for the Taiwan strain and 10 days for the Reunion strain.

### Experimental populations

The experiment followed a 2×3×4 factorial design, each combination being replicated 6–8 times for a total of 187 experimental populations. The three crossed factors were the level of temporal autocorrelation in host abundance (four levels, see below for details), the number of founding females (1, 5, or 20), and the geographic strain (Reunion or Taiwan).

Each experimental population was grown in a plastic tube (diameter 50 mm, length 100 mm). At the beginning of the experiment, each population was initiated with mated female parasitoids. Food for adult parasitoids was provided ad libitum as drops of honey placed on the tube walls. Hosts were provided as 3-mm-diameter patches of *E. kuehniella* eggs glued on paper strips (1 patch ≈ 45 host eggs, see below for the description of experimental treatments of host quantity). Host eggs were previously irradiated, thus preventing their development while maintaining their suitability for the *Trichogramma*. This allowed us to circumvent the internal dynamics of the host–parasitoid system and to manipulate host dynamics according to planned experimental treatments.

Patches of host eggs were exposed 48 h to parasitoids and then put aside until parasitoid emergence. Parasitoid population size at the next generation was estimated by counting the total number of parasitized eggs before emergence. Parasitized eggs turn black when the parasitoid reaches the nymphal stage, that is, after intrahost larval mortality has eventually occurred, so that the number of black eggs directly reflects adult population size at emergence. At the beginning of emergence, fresh patches of host eggs were introduced in the tubes and similarly exposed during 48 h to obtain the next parasitoid generation.

Parasitoid population dynamics were recorded across the first ten generations after introduction. Experimental populations were split into three blocks, which were initiated between September and November 2011. This procedure allowed to buffer potential variations in laboratory conditions, so that repeatable patterns across strains and blocks do not reflect higher-level variations from a shared environment.

### Temporal autocorrelation in host abundance

We manipulated the quantity of host eggs provided at each generation in order to generate either uncorrelated or positively correlated variations in carrying capacity. In the uncorrelated populations, the number of patches of host eggs provided at each generation was drawn from a uniform distribution between 1 and 20 (mean = 10.7, variance = 32.9, *n* = 450 for all generations in uncorrelated populations).

**Figure 2:**
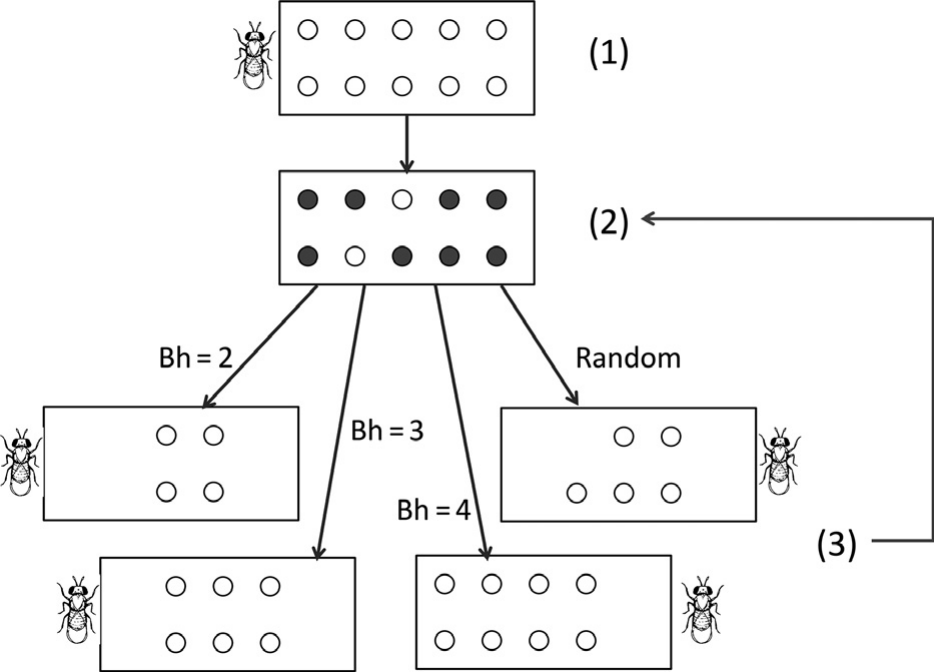
Experimental design. (1) Generation t: Parasitoids are provided with *n* patches of host eggs to parasitize (1 patch = 45 eggs). (2) After parasitism stage, parasitized eggs turn black, *s* patches have survived (white patches). (3) Generation *t* + 1: Parasitoids emerging from parasitized eggs are provided with fresh patches of host eggs. Left: Host reproductive rate *Bh* = 2, 2*s patches are provided; Bottom left: *Bh* = 3, 3*s patches are provided; Bottom right *Bh* = 4, 4*s patches are provided; Right: the number of patches is randomly drawn from a uniform distribution.

In the autocorrelated populations, twenty patches of host eggs were provided for the first generation. For the generations 2–10, the provided number of host eggs, *H*_*t+1*_, was determined from the quantity of nonparasitized eggs at the previous generation *H*_*surv,t*_, multiplied by an artificial reproductive rate *B*_*h*_ (Fig.2). For practical purposes, the maximum number of patches provided was 20, which can be considered as the carrying capacity of host population in absence of parasitoids. To avoid extinction of the system by overexploitation of the host, at least one patch of host eggs was provided at each generation. We tested three values for *B*_*h*_ (2.0, 3.0 and 4.0), in order to obtain a gradient in the dependency of host and parasitoid dynamics (i.e., for *B*_*h*_ = 2, host dynamics are strongly determined by parasitoid abundance, while for *B*_*h*_ = 4, host dynamics are much less affected by parasitism), which also generated a gradient in host autocorrelation (see below, Quantitative differences between treatments).

### Statistical analyses

#### Colonization success

Several populations were not successfully colonized, that is, extinction occurred at the first generation. Colonization failure was considered as a binary variable (0: successfully colonized population; 1: population extinct at the first generation) and was analyzed with generalized linear mixed models. Block was added as a random factor. Fixed effects included the number of founding females and the geographic strain. Host dynamics was not added as an explanatory variable, as these extinctions occurred before the host population could be impacted by parasitism pressure.

#### Quantitative differences between treatments

For each population, the level of autocorrelation was estimated as the autocorrelation coefficient at lag 1 (i.e., the correlation coefficient between successive values of host abundance) calculated over the whole time series. We selected this time lag as it was expected to be the most relevant for effects related to overcompensatory dynamics, that is, mismatch between population size and carrying capacity in successive generations. We nevertheless checked that partial autocorrelations at higher time lags were not significant, that is, there were no residual autocorrelations once the dependence at lag 1 was removed. This was done graphically by drawing the sample partial autocorrelation plot for lag 1, with 95% confidence intervals.

Then, the average number of available host patches, the coefficient of variation in host abundance, and the level of autocorrelation at lag 1 were compared between modalities of host dynamics (four modalities uncorrelated, and the three different values of *B*_*h*_) to characterize the impact of the experimental treatments on host abundance. These variables did not follow classical distributions and thus were analyzed with Kruskal–Wallis tests.

#### Parasitoid establishment dynamics

Parasitoid dynamics were characterized by (1) mean parasitoid population size*,* (2) parasitoid population coefficient of variation (standard deviation of population size across generations divided by mean population size), and (3) probability of extinction within 10 generations. Parasitoid population size and coefficient of variation followed a normal distribution and were analyzed with linear mixed models. Extinction was considered as a binary variable describing the fate of populations at the end of the experiment (0: persisting population; 1: extinct population) and was analyzed with generalized linear mixed models. Block was added as a random factor. The general goodness of fit of models was assessed by checking the marginal pseudo-*R*^2^ of the mixed models (Nakagawa and Schielzeth 2013). Experimental treatment was always a significant predictor of all response variables. However, experimental treatments were found to differ in the average number of host patches, the level of host variability, and the level of autocorrelation in host abundance. Therefore, in order to better understand the respective influence of each of these components on parasitoid dynamics and to achieve a better power of generalization, we included these three predictors as quantitative covariates in the analyses instead of treatment as a factor.

In all three analyses, the number of founding females, the geographic strain, and the number of available host patches were always included as fixed effects. Significance of these fixed effects was estimated by likelihood ratio tests between nested models using maximum-likelihood estimation (for linear models) or Laplace estimation (for generalized linear models). The effect of the structure of environmental variation was tested specifically by comparing the fit of four competing models including as covariates: (1) the coefficient of variation, (2) the coefficient of autocorrelation of host abundance, (3) both variables, or (4) neither. We calculated the Akaike weight of each model, and we retained the 95% confidence set of models for model averaging with standardized predictors (Burnham and Anderson 2002; Nakagawa and Freckleton 2010; Grueber et al. 2011). This method allowed us to estimate the respective influence of the amount of host variability and the structure of this variation on parasitoid establishment dynamics.

## Results

### Colonization failures

Thirty-six populations (of 187) went extinct at the first generation, that is, the founding females failed to produce any offspring. The probability of such colonization failures decreased with the number of founding females (*P* < 0.001) but was not significantly different across geographic strains (*P* = 0.40). As these early extinctions were independent of host dynamics, we did not include the corresponding populations in the following analyses.

### Characterization of host dynamics treatments

The average number of available host patches was highly different across host dynamics treatments (Kruskal–Wallis 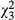 = 62.18, *P* < 0.001). In particular, the average number of host patches was higher in autocorrelated treatments with *B*_*h*_ = 3.0 and *B*_*h*_ = 4.0 than in the uncorrelated treatment and the autocorrelated treatment with *B*_*h*_ = 2.0 (multiple comparisons between *B*_*h*_ = 3.0 or *B*_*h*_ = 4.0 versus uncorrelated treatment or *B*_*h*_ = 2.0, all *P* < 0.003, Figure S1).

The coefficient of variation in host abundance was also highly different between treatments (Kruskal–Wallis 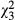 = 34.26, *P* < 0.001). Host abundance was more variable in the uncorrelated treatment and the autocorrelated treatment with *B*_*h*_ = 2.0 than in the autocorrelated treatments with *B*_*h*_ = 3.0 and *B*_*h*_ = 4.0 (multiple comparisons between *B*_*h*_ = 3.0 and *B*_*h*_ = 4.0 versus uncorrelated treatment or *B*_*h*_ = 2.0, all *P* < 0.004, Figure S2).

The temporal autocorrelation coefficient of host abundance at lag 1 was highly different across treatments (Kruskal–Wallis 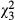 = 54.41, *P* < 0.001). In the uncorrelated treatment, the realized autocorrelation was slightly negative and significantly different from autocorrelation in all autocorrelated treatments (multiple comparisons, all *P* < 0.005). Autocorrelation in host dynamics decreased when *B*_*h*_ increased (multiple comparisons: populations with *B*_*h*_ = 2 significantly different from populations with *B*_*h*_ = 4, *P* = 0.02, marginally different from populations with *B*_*h*_ = 3, *P* = 0.09, Figure S3).

### Parasitoid population size

**Figure 3:**
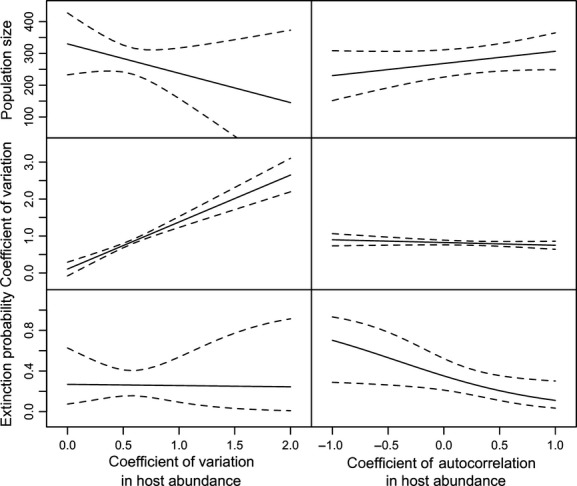
Effect of coefficient of variation (left) and autocorrelation coefficient at lag 1 (right) in host abundance on parasitoid population size (top), the coefficient of variation of parasitoid populations (middle), and the extinction probability of parasitoid populations (bottom). Solid lines: partial effects model fits. Dotted lines: 95% confidence interval.

Parasitoid population size was best predicted by both host variability and autocorrelation in host abundance (Table1). Mean parasitoid population size was negatively correlated with host variability, but positively correlated with temporal autocorrelation in host abundance, that is, populations in a variable environment were on average smaller (Fig.3, top left), but this effect was attenuated if environmental variation was positively autocorrelated (Fig.3, top right). Parasitoid population size was also positively correlated with the mean number of patches of host eggs, that is, habitat size (*P* = 0.009), and with the number of founding females (*P* = 0.002) but was independent of geographic strain (*P* = 0.75).

**Table 1:** Summary results for the model averaging procedure evaluating the respective influence of host variability and host autocorrelation on parasitoid establishment dynamics.

Response variable	Predictors (standardized)	Estimate	Adjusted SE	95% Confidence interval	*P*-value (*z*-test)	Relative importance
Parasitoid population size	Host variability	−93.3	39.8	[−171.3, −15.2]	0.019	0.68
	Host autocorrelation	39.2	19.3	[1.3, 77.1]	0.042	0.82
Coefficient of variation of parasitoid population	Host variability	0.86	0.11	[0.65, 1.08]	<0.001	1
	Host autocorrelation	−0.09	0.04	[−0.17, −0.003]	0.041	0.71
Extinction probability	Host variability	−0.15	1.56	[−3.2, 2.9]	0.92	0.29
	Host autocorrelation	−1.3	0.51	[−2.3, −0.30]	0.011	0.94

### Variability of parasitoid populations

The coefficient of variation of parasitoid populations was highly dependent on host dynamics (Table1): As predicted, populations in a more variable environment were more likely to fluctuate (Fig.3, middle-left) and thus more likely to experience particularly low population sizes, which is expected to increase their extinction risk. In contrast, temporal autocorrelation in host abundance had only a slight negative effect on the variability of parasitoid populations (Fig.3, middle-right). Coefficient of variation was also lower for the Taiwan strain (*P* = 0.04), negatively affected by the number of founding females (*P* = 0.002) and positively affected by the average number of available patches of host eggs (*P* < 0.001), that is, populations founded by a low number of females or experiencing high host abundance were more variable.

### Parasitoid establishment

Extinction probability after the first generation was mostly determined by autocorrelation in host abundance (Table1). As predicted, the autocorrelation coefficient at lag 1 had a negative effect on extinction probability (Fig.3, bottom right): Populations with strong positive autocorrelation in host quantity were found to be more resistant to extinction. Surprisingly, however, host variability had no influence on extinction probability (Fig.3, bottom left). Extinction was also independent of geographic strain (*P* = 0.96), of the number of founding females (*P* = 0.48), and of the average number of host patches (*P* = 0.11).

## Discussion

As is classically expected for introduced populations (Lockwood et al. 2005; Simberloff 2009; Fauvergue et al. 2012), colonization failure was strongly correlated with the number of founding females. Stochastic factors were determinant at the colonization stage, and a higher number of founding females gave a better chance that at least one individual emerged at the next generation. Once initial colonization was achieved, population dynamics were only marginally affected by the geographic strain, thus suggesting a strong influence of demographic rather than genetic processes during establishment phase in the considered experimental system. Among demographic factors, environmental variability was found to affect several components of population dynamics, yet its influence on extinction risk remained moderate. Furthermore, although exposed to significant environmental variation, populations where host abundance was autocorrelated experienced a decreased extinction risk, thus revealing a buffering effect of positive temporal autocorrelation in environmental variation.

### Variable environments harbor both smaller and more variable populations, but do not increase extinction risk

In accordance with classical predictions (Benton et al. 2001, 2002; Drake and Lodge 2004), populations in highly variable environments were on average smaller than populations in more stable environments, even when accounting for differences in mean habitat size. Populations in variable environments were also more variable and thus more likely to experience very small population sizes. These two effects are expected to increase extinction risk, as small and variable populations are most vulnerable to demographic stochasticity. However, we did not find any relationship between the amount of variability in host abundance and extinction risk of parasitoid populations. This surprising result might arise from a reduced level of demographic stochasticity experienced in our experimental populations. Artificial microcosms are usually characterized by more favorable conditions than natural environments, which might level out interindividual differences, lower interindividual variability in reproductive success, and ultimately decrease the impact of demographic stochasticity.

### Lower extinction risk in autocorrelated environments

Theoretical investigations predict that positive autocorrelation in carrying capacity should increase population persistence when population dynamics are overcompensatory (Schwager et al. 2006), which is the case in this experimental system (Vercken et al. 2013). Indeed, when carrying capacity varies randomly, population size (determined by carrying capacity at the previous time step) becomes likely to exceed carrying capacity at some point, which increases the extinction risk in variable environments, whereas smoother variations of carrying capacity are expected when environmental variation is positively autocorrelated.

In addition to this effect related to the mechanistic properties of density-dependent dynamics, one could expect enhanced positive effects of temporal autocorrelation on the persistence of parasitoid populations in particular. Indeed, environmental autocorrelation in our experiment was designed to mimic the natural dynamics of host–parasitoid systems. In this case, temporal autocorrelation in host abundance is likely to have influenced the evolution of the parasitoid population in nature. In particular, when environmental variation is predictable to some extent, selection should favor plastic mechanisms that aim at maximizing one’s fitness in future environmental conditions (Mousseau and Fox 1998; Alpert and Simms 2002; Koops et al. 2003). Maternal effects, such as an environment-dependent reproductive strategy, could have allowed female parasitoids to adjust their offspring quantity and quality according to the prospective state of the environment at the next generation.

### Autocorrelation in the wild: consequences for establishment dynamics in nature

Our experiment evidenced that autocorrelation in environmental variation can counteract several negative effects of environmental variation, such as the decrease in average population size and the increase in population variability. Furthermore, the autocorrelation coefficient was the only predictor of extinction probability (on which it had a negative effect), while the amount of variation in itself was not significant. These findings are likely to shed some light on the establishment dynamics of biological control agents in nature and this for several reasons.

First, agroecosystems are essentially fragmented habitats (Kruess and Tscharntke 1994; With et al. 2002). Therefore, the host pest population size should be limited by the area of a specific crop. Furthermore, as agricultural crops are extensively managed, host-pest populations develop in relatively constant environments. In this context, when a parasitoid is introduced as a biological control agent, host population dynamics are likely to be dominated by fluctuations induced by the host–parasitoid interaction in the midterm, even if the long-term outcome of the interaction is a stable equilibrium (Kidd and Amarasekare 2012). Therefore, establishment success for parasitoids might benefit from their tight interaction with their host dynamics.

On the other hand, because most time series of ecological factors tend to be positively autocorrelated (Steele 1985; Vasseur and Yodzis 2004), fluctuations in host abundance induced by external factors should also positively impact parasitoid establishment. As a consequence, establishment probabilities in variable environments estimated from stochastic models including uncorrelated environmental variation might be underestimated (Grevstad 1999a; Shea and Possingham 2000). Therefore, while correlative and experimental evidence clearly point toward a negative impact of environmental variability on the establishment success of biological agents in the field (see Fauvergue et al. 2012 for a review), our results suggest that background environmental noise might be less decisive than catastrophic events. In particular, as many major disturbances in agroecosystems (e.g., tilling, harvesting, crop rotation, and land-use change) result from management strategies, they are likely to be temporally uncorrelated, which should enhance their negative impact on parasitoid establishment. Our results thus suggest that agroecosystems might prove less favorable to parasitoid establishment than natural environments, in which both internal (i.e., host–parasitoid interactions) and external (e.g., abiotic factors) sources of environmental variability are expected to be positively autocorrelated.

## Conclusions

Whereas negative effects of environmental variation have been reported previously in experimental systems (uncorrelated variation: Belovsky et al. 1999; Drake and Lodge 2004; autocorrelated variation: Pike et al. 2004; Reuman et al. 2008), this study provides the first evidence for a positive influence of temporal autocorrelation in environmental variation on population persistence. These results confirm that, beyond its amplitude, the structure of environmental variation is a key determinant of extinction dynamics that can have opposite effects depending on the ecological context.

As this result emerges from a microcosm experiment, several limitations of the experimental design must be acknowledged. First, the scale of environmental variation investigated here was relatively limited: The host population had a maximum size, which was not affected by random variations, thus constraining the internal and external sources of variation to act on the same scale. This might have contributed to underestimate the influence of random variations on population variability and extinction risk. Second, as mentioned earlier in the discussion, the effects of demographic stochasticity have likely been strongly reduced in our microcosms, thus contributing to underestimate the extinction risk induced by both random and autocorrelated variations in population size.

Therefore, to better understand how internal and external sources of variation may jointly affect host–parasitoid dynamics and population persistence during the establishment phase, studies to come should aim at (1) quantifying the respective influence of background environmental noise, catastrophic events, and host–parasitoid interactions on host variability and (2) investigating the potential interactions between random and autocorrelated sources of variation on parasitoid dynamics.

## Supporting Information

**Figure S1.** Distribution of average number of host patches in experimental populations, in function of experimental treatment on host dynamics. **Figure S2.** Distribution of coefficient of variation in host abundance, in function of experimental treatment on host dynamics. **Figure S3.** Distribution of autocorrelation coefficient in host quantity at lag 1 in experimental populations, in function of experimental treatment on host dynamics.
