# Supplementary material for: Temporal autocorrelation in host density increases establishment success of parasitoids in an experimental system
Source: Ecol Evol. 2015 Jun 18;5(13):2684–93. doi: 10.1002/ece3.1505 (PMC4523363; doi:10.1002/ece3.1505)

Figure S1: Distribution of average number of host patches in experimental populations, in function of experimental treatment on host dynamics.

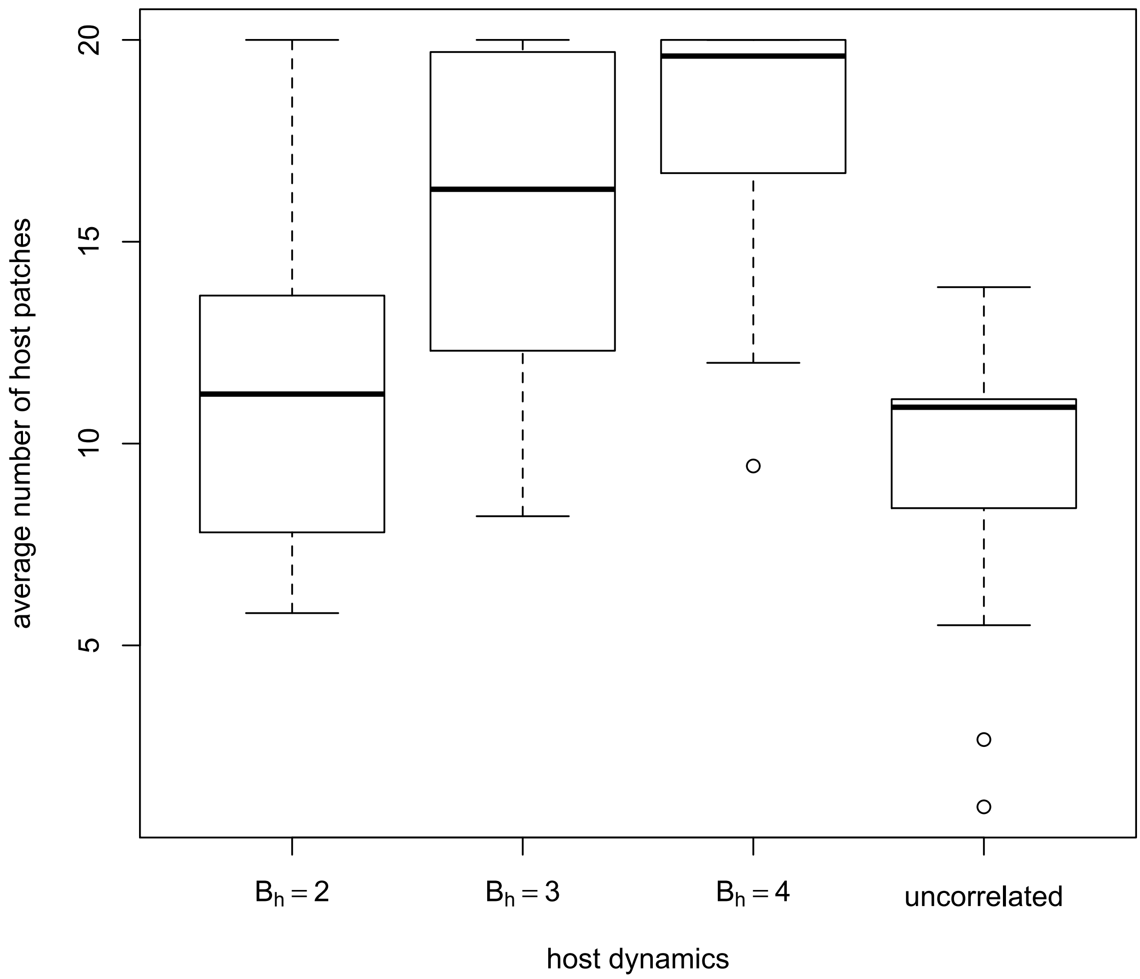

Figure S2: Distribution of coefficient of variation in host abundance, in function of experimental treatment on host dynamics.

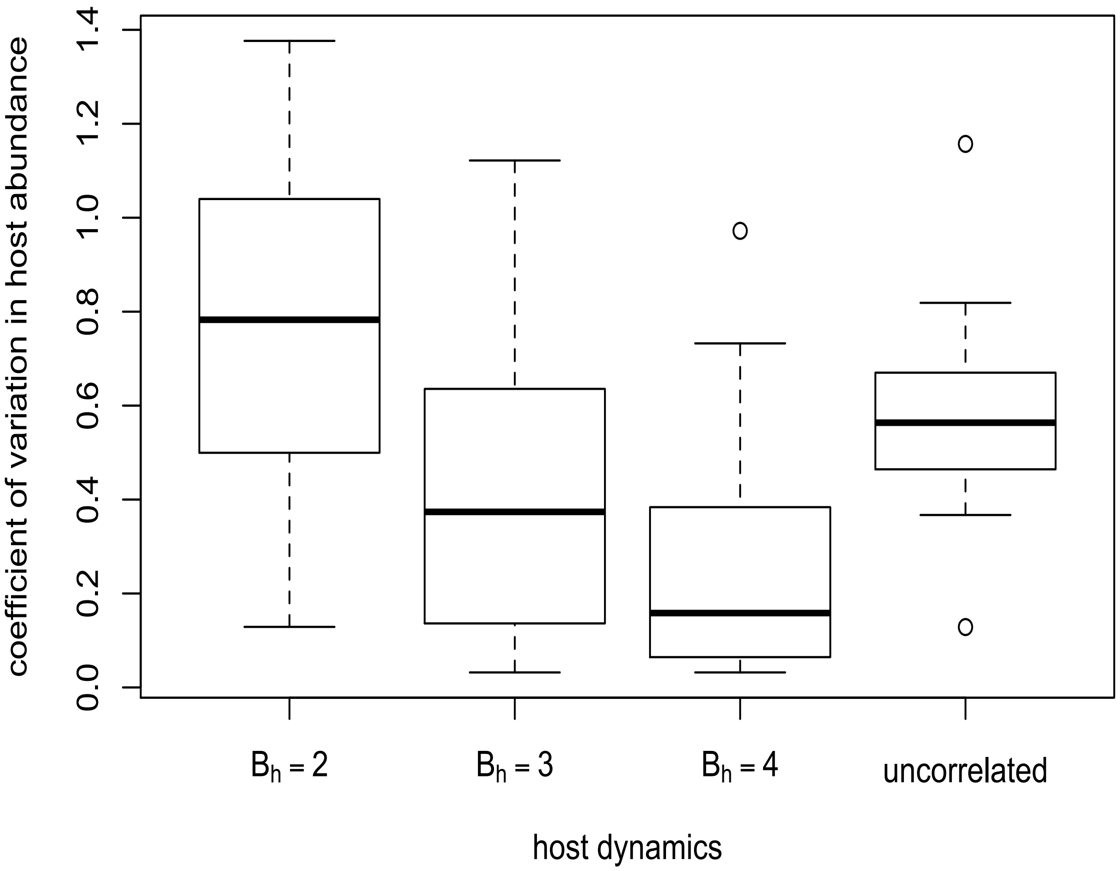

Figure S3: Distribution of autocorrelation coefficient in host quantity at lag 1 in experimental populations, in function of experimental treatment on host dynamics.

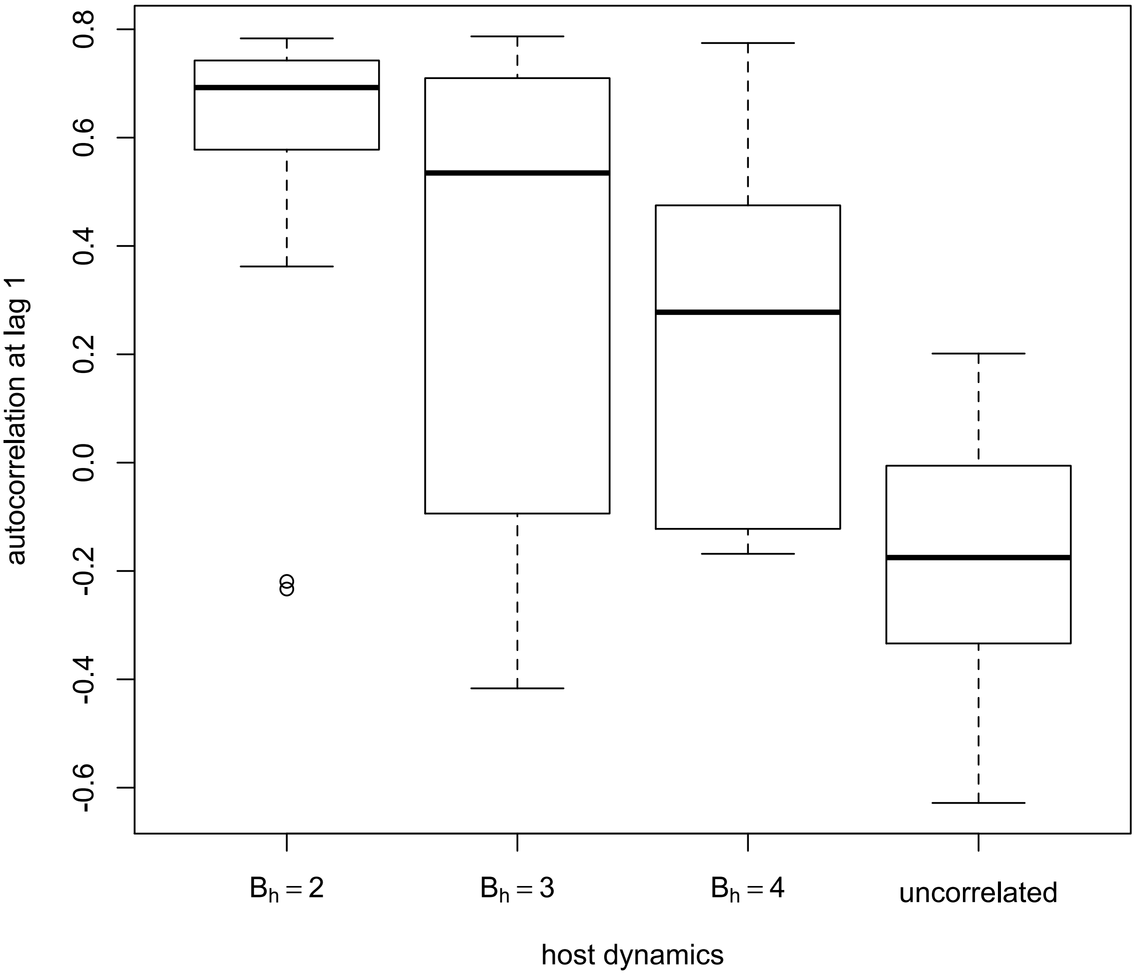

Supplement: Supplementary file 1 [file ece30005-2684-sd1.pdf]
